# Detection Rate and Spectrum of Pathogenic Variations in a Cohort of 83 Patients with Suspected Hereditary Risk of Kidney Cancer

**DOI:** 10.3390/genes14111991

**Published:** 2023-10-25

**Authors:** Zangbéwendé Guy Ouedraogo, Florian Ceruti, Mathis Lepage, Mathilde Gay-Bellile, Nancy Uhrhammer, Flora Ponelle-Chachuat, Yannick Bidet, Maud Privat, Mathias Cavaillé

**Affiliations:** 1 Département d’Oncogénétique, Centre Jean Perrin, 63011 Clermont-Ferrand, France; zgouedraogo@chu-clermontferrand.fr (Z.G.O.); mathis.lepage@clermont.unicancer.fr (M.L.); mathilde.gay-bellile@clermont.unicancer.fr (M.G.-B.); maud.privat@clermont.unicancer.fr (M.P.); 2 Service de Biochimie et Génétique Moléculaire, CHU Clermont-Ferrand, 63000 Clermont-Ferrand, France; 3 Université Clermont Auvergne, CNRS, Inserm, iGReD, 63001 Clermont-Ferrand, France; 4 Service d’Urologie, CHU Gabriel Montpied, 63000 Clermont-Ferrand, France; fceruti@chu-clermontferrand.fr; 5 Université Clermont Auvergne, INSERM, U1240 Imagerie Moléculaire et Stratégies Théranostiques, 63000 Clermont-Ferrand, France

**Keywords:** renal cancer, *CHEK2*, hereditary cancer, cohort, detection rate

## Abstract

Hereditary predisposition to cancer affects about 3–5% of renal cancers. Testing criteria have been proposed in France for genetic testing of non-syndromic renal cancer. Our study explores the detection rates associated with our testing criteria. Using a comprehensive gene panel including 8 genes related to renal cancer and 50 genes related to hereditary predisposition to other cancers, we evaluated the detection rate of pathogenic variants in a cohort of 83 patients with suspected renal cancer predisposition. The detection rate was 7.2% for the renal cancer genes, which was 2.41-fold higher than the estimated 3% proportion of unselected kidney cases with inherited risk. Pathogenic variants in renal cancer genes were observed in 44.5% of syndromic cases, and in 2.7% of non-syndromic cases. Incidental findings were observed in *CHEK2*, *MSH2*, *MUTYH* and *WRN*. *CHEK2* was associated with renal cancer (OR at 7.14; 95% CI 1.74–29.6; *p* < 0.003) in our study in comparison to the gnomAD control population. The detection rate in renal cancer genes was low in non-syndromic cases. Additional causal mechanisms are probably involved, and further research is required to find them. A study of the management of renal cancer risk for *CHEK2* pathogenic variant carriers is needed.

## 1. Introduction

Kidney cancer is among the most common cancers. Worldwide incidence and mortality rates in 2020 were estimated at 431,288 new cases and 179,368 deaths (Globocan 2020). This cancer rarely arises before the age of 40. Men are more frequently affected than women, with the sex ratio ranging from 1.7 to 2.3 in Europe. Other well-established risk factors include cigarette smoking, obesity, hypertension, and exposure to chemicals such as trichloroethylene, while physical activity and consumption of fruits and vegetables reduce the risk [1,2,3,4,5,6,7,8,9,10]. 

In addition to environmental and lifestyle factors, hereditary predisposition to kidney tumours occurs in approximately 3% of cases [11]. Up to 17 genes have been identified as conferring a hereditary risk of kidney cancer [12,13,14,15,16,17,18]. The major genes are *VHL* (OMIM * 608537), *FH* (OMIM * 136850), *MET* (OMIM * 164860), and *FLCN* (OMIM * 607273), involved, respectively, in Von Hippel–Lindau (VHL) disease, hereditary leiomyomatosis and renal cell carcinoma (HLRCC), hereditary papillary renal cell carcinoma, and Birt–Hogg–Dubé (BHD) syndromes. Other genes may also be involved, including *BAP1* (OMIM * 603089), *SDHB* (OMIM * 185470), *PTEN* (OMIM * 601728), *TSC1* (OMIM * 605284) and *TSC2* (OMIM * 191092). 

Different guidelines have been proposed worldwide for genetic testing for suspected hereditary risk of kidney cancer, including those of the National Comprehensive Cancer Network (NCCN) in the USA, the Predispositions aux tumeurs du Rein (PREDIR) network of the Institut National du Cancer (INCa), or the Comité de cancérologie de l’Association Française d’Urologie (ccAFU) in France [17,18]. Our department used an early version of PREDIR guidelines, with inclusion age of <50 years (and not <46 years) for isolated clear cell cancers (Table 1).

Some studies have reported the diagnostic rate of genetic testing for inherited kidney cancer risk assessment. Carlo et al. evaluated the prevalence of mutations and their spectrum in patients with advanced renal cell carcinoma (RCC) in a US cohort study that included 254 patients with advanced RCC, unselected for inherited cancer risk factors [15]. Germline variants detected via exon sequencing of 76 genes associated with cancer predisposition were analyzed using panel sequencing. Germline mutations were identified in 41 (16.1%) patients, and mutations in RCC-associated genes (*FH*, *BAP1*, *VHL*, *MET*, *SDHA*, and *SDHB*) in 14 (5.5%) [15]. In a Danish cohort including 49 patients from 48 families with a suspected hereditary risk of renal cancer, Christensen et al. found no pathogenic variants in RCC causative genes (*VHL*, *FH*, *FLCN*, *MET, BAP1*, *SDHB)* and one pathogenic variant in MITF, a putative RCC gene [16]. More recently, in a monocentric Chinese cohort of 123 unselected RCC cases, Feng et al. found a molecular diagnostic yield of 7.3% through screening of 14 RCC-associated genes [19]. There is not yet any study reporting the yield of screening for pathogenic variants in renal cell cancer-associated genes in persons with a suspected hereditary risk of renal cell cancer according to the criteria for referring patients for genetic testing in France. Our study constitutes the largest current European cohort.

We conducted a retrospective cohort study of patients tested in our laboratory. The primary objective was to evaluate the detection rate of pathogenic or likely pathogenic variants in a next-generation sequencing (NGS) panel for kidney cancer predisposition. Our secondary objective was to evaluate the detection rate of pathogenic or likely pathogenic variants using a larger panel of genes implicated in other cancer-predisposing syndromes and not known to be associated with a risk of kidney cancer.

## 2. Materials and Methods

### 2.1. Study Population

Patients with a personal or family history of kidney cancer or with a kidney cancer risk syndrome undergoing genetic testing in the oncogenetics department of Centre Jean Perrin (France) from 2015 to 2022 were eligible (Table 1). Relatives of cases with an identified pathogenic variant were excluded. Patients who did not give written consent for the use of their data for research purposes were excluded. 

Eighty-three patients were included in the analysis, and were classified by type of indication. A syndromic indication was defined as predisposition to renal cancer with extra-renal syndromic manifestations. A non-syndromic indication was diagnosed when no extra-renal manifestations associated with syndromic forms were detected. Non-syndromic indications were divided into groups A, B, and C, as depicted in Table 1.

### 2.2. Genes Included in the NGS Panels

Patients suspected of having an inherited risk of renal cancer were advised to undergo genetic testing using an oriented panel of 8 genes involved in hereditary renal cancer risk, or an extended panel including 58 genes of predisposition to renal cancer as well as other known hereditary cancer syndromes (Table 2). In some rare cases, fewer genes were explored because of a clinical presentation highly suggestive of a specific syndrome (*VHL*, *FLCN*). One case was explored using whole-exome sequencing, as recommended by a multidisciplinary medical meeting. The number of individuals for whom each panel or gene-oriented test was performed is presented in Table 3.

### 2.3. Next Generation Panel Sequencing

Samples of peripheral blood were used for genomic DNA extraction. Sonic fragmentation of DNA was performed using a Bioruptor instrument (Diagenode). Kapa HTP library preparation and SeqCap EZ Choice probes and reagents (Roche) were used for library preparation and capture. Quality of fragmentation, library, and capture were controlled with a Bioanalyzer 2100 instrument (Agilent). Sequencing was performed with a Miseq Instrument (Illumina) using a Miseq v2 kit (300 cycles), or with a NextSeq 500/550 Instrument using a NextSeq v2 kit (300 cycles). Analysis of exons 11 to 15 of PMS2 and exons 1, 13, and 14 of SDHA and a quantitative analysis of WRN exon 10 were not performed due to high identity with paralogous genes. All exons of the remaining genes were sequenced, including intron/exon junctions up to position −20 and +20, as well as the E1′ cryptic exon in *VHL* (NM_001354723.2). The sensitivity of sequencing was 100% and specificity > 99% for single-nucleotide variant or CNV analyses.

### 2.4. Bio-Informatic Analyses

After de-multiplexing using bcl2fastq2 Conversion Software (Illumina), sequence alignment was performed with University of California Santa Cruz human genome reference build 19, using a Burrows–Wheeler Aligner. A Genome Analysis Toolkit (GATK) and PICARD tools were used for base quality score recalibration (BaseRecalibrator) and realignment (RealignerTargetCreator, IndelRealigner), as recommended by Eurogentest guidelines [20]. The variant calling step used GATK HaplotypeCaller and annotation the EnsemblVariantEffectPredictor. Copy number variation was analyzed using ExomeDepth. Variants were filtered for quality score ≥30, depth ≥30x, and presence in ≥20% of reads.

### 2.5. Variant Interpretation

Variant interpretation used ALAMUT (Interactive Bio-Software) and Mobidetails, with splice site analysis tools (SPIP, SpliceAI), protein function prediction tools (SIFT, Polyphen 2.0), and links to relevant databases (GnomAD, ClinVar, Leiden Open Variation Database [LOVD], and other syndrome-specific databases). Variants were classified according to the American College of Medical Genetics (ACMG) recommendations [21]. Only pathogenic (class 5) variants and probably pathogenic (class 4) variants were reported.

### 2.6. Complementary Analyses

All class 5 or class 4 variants were confirmed on a second independent sample using a different method. Sanger sequencing for confirmation was performed using a 3500xl instrument and BigDye terminator kit 3.1 (Applied Biosystems). Electrophoregraphs were analyzed with Seqman software (DNASTAR). Copy number variation (CNV) was confirmed using quantitative multiplex PCR of short fragments (QMPSF), interpreted using GeneMapper software (Applied Biosystems). For splice variants, PAXgene tubes were used for blood sampling, and total RNA was extracted using PAXgene Blood RNA Kit (Qiagen). Total RNA was reverse-transcribed using a high-capacity RNA to cDNA kit (Applied Biosystems) before capture of target sequences and NGS analysis. Relevant exons with insufficient coverage depth (<30x) were re-sequenced via the Sanger method, if relevant.

### 2.7. Statistical Methods

The pathogenic variant detection rate was defined as the proportion of class 4 and class 5 variants in screened individuals, using a defined panel. Allele frequencies in the cohort were compared with allele frequencies in a general population obtained from the gnomAD Exomes version 2.1.1 database. Known pathogenic and likely pathogenic variants were collapsed by gene, and burden tests performed among the cohort vs. the general population of the gnomAD database; odds ratios (ORs) were computed. 

The *p*-value for the test of significance was calculated according to Sheskin [22]. A standard normal deviation (z-value) is calculated as ln(OR)/SE{ln(OR)}, and the *p*-value is the area of the normal distribution that falls outside ±z. Within a two-sided Fisher’s exact test, *p* < 0.05 was estimated as statistically significant [22].

## 3. Results

### 3.1. Demographics of the Cohort

The cohort included 83 unrelated patients with a median age of 48 years (range, 22–88 years). Thirty-four (41%) were females, and the sex ratio was 1.44 males/females (Table 4). Seventy-five patients presented a personal history of renal cancer. Nine individuals were included because they were diagnosed with a syndromic form of renal cancer (BHD (n = 5), VHL (n = 3), and HLRCC (n = 1)). Eight cases did not present a personal history of renal cancer. Seventy-four individuals were diagnosed with non-syndromic renal cancer, with group A consisting of individuals who presented with clear cell RCC at age ≤50 (n = 43) or bilateral clear cell RCC (n = 5); group B included nineteen individuals with papillary renal cancer (n = 5), chromophobe renal cancer (n = 4), tubular-papillary renal cancer (n = 4), oncocytic hybrid tumour (n = 1), mixed component tumour (n = 3), and with multiple histological kidney cancers (n = 2); group C included patients with familial kidney cancer (n = 7). 

### 3.2. Diagnostic Rate and Cross-Cohort Spectrum of Pathogenic Genomic Variations

Genetic testing of the 83 cases detected 11 pathogenic variants and 1 likely pathogenic variant, including incidental findings, in 11 individuals (13.25%) (Table 5). All variants were monoallelic. The diagnostic rate in genes involved in hereditary predisposition to renal cancer was 7.23% (6/83); six pathogenic variants were detected in four of the eight genes of the kidney panel. 

The diagnostic rate in renal cancer genes was 44.44% (4/9) in syndromic individuals and 2.7% (2/74) in non-syndromic individuals, with pathogenic variants in *PTEN* and *FH* in two patients without clinical features of the associated syndromes (Table 5). Syndromic cases represented 11% of the cohort, and 67% of those positive for a renal cancer-causing variant.

Gene-oriented testing (n = 7) found a *FLCN* pathogenic variant detection in two of three cases with BHD syndrome and a *VHL* pathogenic variant in one of three VHL disease-presenting individuals (Table 6). The kidney panel, performed for 26 non-syndromic cases, did not detect any pathogenic or likely pathogenic variants.

For the 49 individuals tested with the cancer panel, we detected a *BHD* pathogenic variant in a BHD syndrome-presenting individual and an *FH* pathogenic variant in a non-syndromic individual (Table 6). Consequently, the cancer panel diagnostic rate in renal cancer genes was estimated to be 4.08% (2/49). This panel provided incidental discoveries of four pathogenic and one likely pathogenic variant in other cancer-associated genes in five individuals, all in non-syndromic cases (Table 6). 

Regarding the other cancer-associated genes, the cancer panel and exome detected six incidental variants in five patients (10%), in four different genes: one pathogenic and one likely pathogenic variant in *CHEK2*, two pathogenic variants in *MUTYH*, and one each in *MSH2* and *WRN* (Table 5). As previously reported, the *PTEN* variant and a *MUTYH* variant were carried by the same individual [23].

Four variants of unknown significance were found in the cohort (4/83; 5%). The variant *WRN* (NM_000553.4) c.2371T>G; p.(Cys791Gly) was found in a BHD patient. Other variants (*BRCA2* (c.5900A>G; p.(Lys1967Arg)), *ATM* (c.1810C>T; p.(Pro604Ser)) and *MET* (c.2962C>T; p.(Arg988Cys)) were found in non-syndromic patients (3/74; 4%) with clear cell renal cancer before 50 years of age.

## 4. Discussion

This cohort of 83 individuals suspected of having a genetic risk of kidney cancer included 74 non-syndromic cases and 9 syndromic cases. The diagnostic rate in renal cancer genes was 44% (4/9) in syndromic cases and 2.7% in non-syndromic cases. The global cohort diagnostic rate was 7.2% (6/83), representing a 2.4-fold enrichment in inherited risk in comparison with the estimated prevalence of genetic predisposition to renal cancer in patients with this cancer (3%). However, only the syndromic patients presenting renal cancer with extra-renal manifestations seemed to be associated with a high molecular diagnostic yield (4/9, nearly 50%). The prevalence of hereditary predisposition to cancer in non-syndromic forms is low in our study (2/74, 2.7%), comparable to that presented in the Danish cohort (2.17%), and lower than in the Chinese cohort or in the unselected advanced renal cancer cohort (5.5–7.3%) [15,19].

Thus, the current analysis criteria for genetic testing used in the European cohorts (Danish and ours), based on age, number of cancers, histological type, and family history, are inefficient for targeting non-syndromic patients at risk. In our study, the age of occurrence of the two non-syndromic carriers of a mutation in a known renal cancer gene was very young (28 and 31 years of age). The age threshold for constitutional genetic analysis of patients with a non-syndromic form of cancer could be reduced. Further studies using a larger cohort could help answer this hypothesis. 

The difference observed between European and Chinese cohorts could be linked to an ethnic effect, or also to a reduced cohort size in these different studies.

The variants we found in non-syndromic patients (*FH*, *PTEN*) are usually identified in patients with syndromic forms of cancer, which was not the case in our study. The patient with an *FH* pathogenic variant was a woman who presented unilateral unclassified renal cancer at 28 years of age. This patient did not present cutaneous or gynecological features of HLRCC. It should be noted that FH immunohistochemistry had not been performed on the tumour sample, which could have oriented the diagnosis for this syndrome. 

The patient with the *PTEN* pathogenic variant presented an atypical form of Cowden syndrome, not fulfilling the clinical criteria for analysis, hence the exploration using exome sequencing and categorization of a non-syndromic form [23].

Most of the non-syndromic patients, despite the presence of testing criteria, did not carry a causal variant in a known predisposing gene. This could suggest the involvement of non-genetic risk factors or the presence of other genetic factors that remain to be discovered. In this context, more extensive panel analyses may be interesting in the search for candidate genes. In our study, a broad panel of 58 genes was performed in 49 patients, and whole-exome sequencing in 1 patient. Six additional heterozygous pathogenic incidental findings were found in *CHEK2*, *MSH2*, *MUTYH* (n = 2), and *WRN* in five patients (6/50, 10%). Thus, including incidental findings, 11 individuals from 83 of the cohort (13.25%) harboured at least one pathogenic or likely pathogenic variant. This proportion is comparable to the 10.57% found in a Chinese cohort of 123 subjects [19]. The genes targeted in the testing and the incidental findings found, were, however, different between the Chinese study (*FANCM*, *FANCI*) and ours (*CHEK2*, *MSH2*, *MUTYH*, *WRN*).

*CHEK2* encodes a serine-threonine kinase which is involved in various pathways, including cell cycle and apoptosis regulation, as well as DNA repair. Loss-of-function variants of this tumour suppressor gene confer a moderate risk of breast cancer, in addition to a risk of colorectal, prostate, and thyroid cancer [24]. Several studies show an association between pathogenic variants in *CHEK2* and the hereditary risk of kidney cancer [24,25,26,27,28,29]. Carlo et al. found an odds ratio of 3.0 (95% CI, 1.3–5.8; *p* = 0.003) from a cohort of 257 patients with advanced renal cancer; Zlowocka-Perlowska et al. found an OR of 2.5 (95% CI, 1.5–4.1; *p* = 0.0003) from a cohort of 835 unselected renal cancer patients; and Bychkovsky et al. found an OR of 2.57 (95% CI, 1.75–3.68; *p* < 0.001) in a cohort of 3783 *CHEK2* mutation carriers [26,27,29]. Using the same data of general population frequency of pathogenic variants in *CHEK2* as those of Carlo et al. 2018, a significantly higher frequency of *CHEK2* (2/49) pathogenic and likely pathogenic variants was found in our cohort (OR at 7.14; 95% CI 1.74–29.6; *p* < 0.003). This is consistent with previous associations, although the confidence interval is wide in our study due to small patient numbers. Thus, *CHEK2* may be associated with renal cancer risk. 

At present, *CHEK2* is not included in renal cancer panel genes, and no management is recommended for these patients. In Europe and North America, the lifetime risk of renal cancer is estimated between 1.3% and 1.8% [30]. An OR associated with *CHEK2* ranges between 2% and 3%, according to studies with a restricted confidence interval, which corresponds to a lifetime renal cancer risk between 2.6–3.6% and 3.9–5.4%. These degrees of risk do not seem sufficient to propose systematic screening and therefore do not argue in favour of the integration of *CHEK2* in renal gene panels in an oncogenetic diagnostic framework at the moment. Further studies taking into consideration the type of mutation, the familial history of renal cancer, and non-genetic risk factors in addition to *CHEK2* genotype could refine the degree of risk for these patients, and thus better define the management of *CHEK2* mutations for predisposition to renal cancer. 

*MSH2* is a mismatch repair (MMR) gene involved in Lynch syndrome in monoallelic carriers. This syndrome predisposes individuals to a high risk of colon and endometrial cancers, and other cancers to a lesser degree, including urothelial cancers of the urinary excretory tract [31,32]. The involvement of MMR genes in predisposition to kidney cancer is controversial [33,34,35]. However, a recent meta-analysis of MMR germline mutation carriers compared to unaffected participants showed a significantly higher relative risk (RR) of developing kidney cancer (RR: 3.97, 95% CI: 1.23, 12.81; *p* < 0.01) [36]. We observed the c.942+3A>T variant in a patient with papillary type I renal cancer and Lieberkuhnian adenocarcinoma of the caecum. This variant induces pathogenic loss of exon 5 [37]. However, MMR protein expression analysis using immunohistochemistry did not show loss of protein expression in the renal cancer sample, unlike the digestive tumour sample, which presented microsatellite instability and loss of MSH2/MSH6 protein expression. This result does not suggest the involvement of *MSH2* in renal tumourigenesis in this patient.

The *WRN* gene encodes for a protein with DNA helicase and exonuclease activities. It is associated with Werner syndrome, an autosomal recessive inherited syndrome characterized by premature ageing. It also predisposes to some malignancies, including sarcoma, but not to renal carcinoma [38,39]. Monoallelic carriers do not seem to show any strong predisposition to cancer, although some studies suggest a small risk of breast cancer (OR 2.0) [40]. *MUTYH* is a base excision repair gene. Biallelic pathogenic variant carriers present *MUTYH*-associated polyposis (MAP) which predisposes to colorectal cancers and other cancers including duodenal and gastric cancers [41,42]. Zeng et al. (2022) found an association between biallelic carriers and renal cancer, with an OR of 32.28 [95% CI, 6.40–162.73] in a population of 214,020 participants from different cohorts [43]. Monoallelic carriers, very common in the general population (1 to 2%), do not seem to be predisposed to moderate or high risk of cancer. Thus, the identification of incident variants in these two genes (*WRN*, *MUTYH*) that are common using next-generation sequencing is probably not associated with a hereditary risk of kidney cancer.

## 5. Conclusions

The detection rate in renal cancer predisposition genes is low in patients with non-syndromic analysis indications. Current clinical criteria are not effective in selecting patients at risk. Additional causal mechanisms are probably involved, including genetic and non-genetic factors, and further research is required to find them using whole-exome or whole-genome sequencing. *CHEK2* seems to be associated with renal cancer risk with low penetrance. This is not sufficient to propose a systematic cancer screening in carriers, or to include *CHEK2* in kidney cancer gene panels. Further studies taking into consideration the type of mutation, the familial history, and the non-genetic risk factors could refine the degree of risk and interest in *CHEK2* in medical practice for renal predisposition to cancer.

## Figures and Tables

**Table 1:** Genetic testing criteria of hereditary predisposition to kidney cancer.

Indication type	Description	
Syndromic	Von Hippel/Lindau disease	
	Birt/Hogg/Dubé syndrome	
	Hereditary leiomyomatosis and renal cell cancer	
	Tuberous sclerosis complex	
Non-syndromic	Clear cell RCC with any of the following criteria: age of diagnosis <50, bilateral or multiple	Group A
	Renal cancer with a histological subtype other than clear cell carcinoma	Group B
	Family history of kidney cancer: kidney cancer and ≥1 close relative with a kidney cancer	Group C

**Table 2:** **Gene panel composition**. The kidney panel included eight genes (in the red limited area) involved in renal cancer risk inheritance. The cancer panel included the kidney panel and 50 genes involved in predisposition to other cancers.

*BAP1*	*BRIP1*	*MEN1*	*SDHC*	
*FH*	*CASR*	*MLH1*	*SDHD*	
*FLCN*	*CDC73*	*MSH2*	*SMAD4*	
*MET*	*CDH1*	*MSH6*	*SMARCB1*	
*MITF*	*CDK4*	*MUTYH*	*STK11*	
*PTEN*	*CDKN1B*	*NF1*	*TMEM127*	
*SDHB*	*CDKN2A*	*NF2*	*TP53*	
*VHL*	*CHEK2*	*PALB2*	*WRN*	
*AIP*	*CTNNA1*	*POLD1*		
*AP2S1*	*DICER1*	*POLE*		
*APC*	*EPAS1*	*PRKAR1A*		
*ATM*	*EPCAM*	*RAD51C*		
*AXIN2*	*GNA11*	*RAD51D*		
*BMPR1A*	*LMNA*	*RET*		
*BRCA1*	*MAX*	*SDHA*		
*BRCA2*	*MDH2*	*SDHAF2*		

**Table 3:** **Molecular analysis explorations.** Oriented analysis included one to eight genes. VHL: Von Hippel–Lindau disease, HLRCC: hereditary leiomyomatosis and renal cell carcinoma, BHD: Birt–Hogg–Dubé.

Indication type	Syndrome or Group	Exome	Cancer panel	Kidney panel	VHL oriented	FLCN oriented	SDH and VHL oriented	Total
Syndromic	VHL				3			3
	BHD		3			2		5
	HLRCC		1					1
Non-syndromic	Group A	1	26	20			1	48
	Group B		13	5		1		19
	Group C		6	1				7
Total		1	49	26	3	3	1	83

**Table 4:** Demographic characteristics of the cohort.

Section	Item	Number	%
Cohort composition	Subjects	83	100
Age	Median at risk diagnosis	45	
	Median at testing	48	
	Range at risk diagnosis	21-73	
	Range at testing	22-88	
Sex	Female	34	41
	Male	49	59
Medical history of renal cancer	Personal history of renal cancer	75	90.36
	Clear cell renal cell carcinoma at >50 years and a familial history of renal cancer	7	8.43
Personal tumour histologic subtype	Clear cell	55	66.27
	Papillary	5	6.024
	Tubulo-papillary	4	4.82
	Chromophobe	5	6.024
	Hybrid oncocytic tumour	1	1.20
	Pluritissular renal cancer	3	3.61
	Multiple renal cell carcinoma	2	2.41
Syndromes with hereditary risk of renal cancer		9	10.84
	BHD	5	6.024
	HLRCC	1	1.20
	VHL	3	3.61

**Table 5:** Description of cases in which pathogenic or likely pathogenic variants were detected. The pathogenic variants in the kidney panel are outlined in red. Outside of this area are the incidentally-discovered variants, i.e., variants in genes other than the kidney panel genes. All variants were heterozygous.

Test purpose	Indication	Group	IA	Testing panel	Variant	
					Annotation	Class
Syndromic	BHD	/	1	*FLCN*	FLCN(NM_144997.7):c.755dup; [p.(Cys253Valfs*39)]	5
	BHD	/	2	*FLCN*	***FLCN(NM_144997.7):c.619-1G>A; [p.?]***	5
	BHD	/	3	Cancer panel	***FLCN(NM_144997.7):c.(?_-1)_(249+1_250-1)del; [p.?]***	5
	VHL	/	4	*VHL*	***VHL(NM_000551.4):c.341-2A>C; [p.?]***	5
Non Syndromic	Clear cell renal cell carcinoma at 28 years	A	5	Cancer panel	***FH (NM_000143.4): c.1118A>G; [p.(Asn373Ser)]***	5
	Metachrone (multiple) kidney cancers	A	6	WES	PTEN (NM_000314.8): c.1003C>T; [p.(Arg335*)]	5
					***MUTYH (NM_001048174.2):c.924+3A>C; [p.?]***	5
	Clear cell renal cell carcinoma at 43 years	A	7	Cancer panel	***MUTYH(NM_001048174.2):c.849+3A>C; [p.?]***	5
	Association of clear cell renal cell carcinoma to a rectal adenocarcinoma and CML with a familial history of several cancers	A	8	Cancer panel	***CHEK2(NM_007194.4):c.349A>G; [p.(Arg117Gly)]***	4
	Bilateral clear cell renal carcinoma	A	9	Cancer panel	***WRN(NM_000553.6):c.(96+1_97-1)_(724+1_725-1)del; [p.?]***	5
	papillary type I renal cell carcinoma	B	10	Cancer panel	CHEK2(NM_007194.4):c.1100del; [p.(Thr367Metfs*15)]	5
	Multiple cancers (papillary type I renal cell carcinoma + lieberkuhnian carcinoma of the caecum)	B	11	Cancer panel	***MSH2(NM_000251.3):c.942+3A>T; [p.?]***	5

**Table 6:** Diagnostic rate and incidental findings in molecular explorations.

Results	Exome	Cancer Panel	Kidney Panel	*VHL*	*FLCN*	*SDHB/VHL*	Overall
Screened individuals	1	49	26	3	3	1	83
Number of pathogenic variants in RC genes	1	2	0	1	2	0	6
Number of incidental variants	1	5	Not applicable	3	3	0	6
Renal cancer genes detection rate	100%	4%	0%	33.3%	66.7%	0%	7.2%
Incidental variant detection rate	100%	10.2%	Not applicable	Not applicable	Not applicable	Not applicable	7.2%

## Data Availability

Due to institutional regulation, patients’ personal data cannot be made publicly available. However, we can respond to acceptable requests that can be sent to the corresponding authors.
